# Roles of *Syzygium* in Anti-Cholinesterase, Anti-Diabetic, Anti-Inflammatory, and Antioxidant: From Alzheimer’s Perspective

**DOI:** 10.3390/plants11111476

**Published:** 2022-05-31

**Authors:** Mira Syahfriena Amir Rawa, Mohd Khairul Nizam Mazlan, Rosliza Ahmad, Toshihiko Nogawa, Habibah A. Wahab

**Affiliations:** 1 Collaborative Laboratory of Herbal Standardization (CHEST), School of Pharmaceutical Sciences, Universiti Sains Malaysia, Bayan Lepas 11900, Malaysia; mirasyah@usm.my (M.S.A.R.); khairulnizamkmk@usm.my (M.K.N.M.); rosliza_ahmad@usm.my (R.A.); 2 USM-RIKEN Interdisciplinary Collaboration for Advanced Sciences (URICAS), Universiti Sains Malaysia, Gelugor 11800, Malaysia; nogawat@riken.jp; 3 Molecular Structure Characterization Unit, RIKEN Center for Sustainable Resource Science, Technology Platform Division, 2-1 Hirosawa, Saitama 351-0198, Japan

**Keywords:** *Syzygium*, medicinal plants, Alzheimer’s disease, multi-target, neuroprotection, anti-cholinesterase, anti-diabetic, anti-inflammatory, antioxidant

## Abstract

Alzheimer’s disease (AD) causes progressive memory loss and cognitive dysfunction. It is triggered by multifaceted burdens such as cholinergic toxicity, insulin resistance, neuroinflammation, and oxidative stress. *Syzygium* plants are ethnomedicinally used in treating inflammation, diabetes, as well as memory impairment. They are rich in antioxidant phenolic compounds, which can be multi-target neuroprotective agents against AD. This review attempts to review the pharmacological importance of the *Syzygium* genus in neuroprotection, focusing on anti-cholinesterase, anti-diabetic, anti-inflammatory, and antioxidant properties. Articles published in bibliographic databases within recent years relevant to neuroprotection were reviewed. About 10 species were examined for their anti-cholinesterase capacity. Most studies were conducted in the form of extracts rather than compounds. *Syzygium aromaticum* (particularly its essential oil and eugenol component) represents the most studied species owing to its economic significance in food and therapy. The molecular mechanisms of *Syzygium* species in neuroprotection include the inhibition of AChE to correct cholinergic transmission, suppression of pro-inflammatory mediators, oxidative stress markers, RIS production, enhancement of antioxidant enzymes, the restoration of brain ions homeostasis, the inhibition of microglial invasion, the modulation of ß-cell insulin release, the enhancement of lipid accumulation, glucose uptake, and adiponectin secretion via the activation of the insulin signaling pathway. Additional efforts are warranted to explore less studied species, including the Australian and Western *Syzygium* species. The effectiveness of the *Syzygium* genus in neuroprotective responses is markedly established, but further compound isolation, in silico, and clinical studies are demanded.

## 1. Introduction

Alzheimer’s disease (AD) is the most prevalent type of dementia worldwide, constituting 70–80% of cases, primarily among the elderly [1]. This irreversible neurodegenerative disorder progressively impairs memory and other cognitive functions. It is characterized by the formation of neurofibrillary tangles and amyloid ß (Aß) protein plaques in the brain, leading to complete brain failure and eventually death [2]. The aggregation of hyperphosphorylated tau microtubule-associated proteins is responsible for the abnormal intracellular tangles, whereas the discharge of Aß peptide produced by the cleavage of the amyloid precursor protein (APP) results in the extracellular senile plaques. Family heredity and genetic factors can play a definite role in AD. However, aging still becomes one of the leading risk factors, although AD is not a normal part of aging [3].

The underlying mechanism of AD remains to be elucidated, but the evidence gathered through clinical and multidisciplinary studies has depicted AD as multifactorial [4]. Both early-onset (a rare form) and late-onset (the most prevalent form of AD) manifest multifaceted toxicity associated with their multifactorial nature [2]. More than 20 various pathological events have been described to give rise to multifactorial AD, including well-known amyloid toxicity, tau burden, cholinergic toxicity, oxidative stress, the role of cholesterol, insulin resistance, and diabetes [4]. Metal ion toxicity, neurovascular toxicity, α-synuclein mediated toxicity, membrane toxicity, biomolecular damage, immune outrage, glucose hypometabolism, and lymphatic dysfunction can also contribute to AD progression [4]. These pathological events are highly connected, leading from one cause to another. The currently approved drugs can only delay the progression as they may target certain pathological burdens while the others remain accelerating.

Natural products are a convincing starting point for exploring neuroprotective agents. Galantamine is currently an AD licensed drug derived from a natural product that helps to alleviate cognitive symptoms [5]. Huperzine A is among the alkaloid-based lead candidates for anti-AD, originating from the herb *Huperzia serrata* [5]. Natural products, especially from medicinal plants, provide inexhaustible sources of bioactive compounds with diverse biological effects if used appropriately. They are widely used in rural communities and studied for biological potentials, considering their cheaper option and accessibility. For example, *Ginkgo biloba* is a Chinese herbal medicine extensively investigated in AD preclinical and clinical trials [6]. It is rich in flavonoids and terpenoids responsible for anti-inflammatory, antioxidant, anti-apoptosis, and protection against Aß aggregation and mitochondrial dysfunction [6]. Multiple large-scale clinical trials are still entailed, but it is noteworthy that *G. biloba* is a versatile multitarget agent against AD.

In principle, multifactorial AD entails remedies that can deliver multiple effects to slow down the progression effectively. Medicinal plants meet the criteria to provide diverse bioactive constituents with low toxicity either in extract or compound form. *Syzygium* is a relatively understated plant genus; it is a functional food that has long been used as complementary medicine but is not fully utilized [7,8]. *Syzygium* species exert various biological activities, including antioxidant, anti-inflammatory, anti-diabetic, and anti-cholinesterase, and are deemed suitable as neuroprotective agents [7,8]. This review aimed to summarize the neuroprotective effects of *Syzygium* species for the first time, focusing on anti-cholinesterase, anti-diabetic, anti-inflammatory, and antioxidant properties. Their current trend, future direction, and limitations as an alternative source for the anti-AD remedies were discussed.

## 2. Multifactorial AD and Multitarget Agents as a Strategy for Neuroprotection

The multifactorial nature of AD has given rise to various hypotheses based on the pathophysiological and molecular observations in the AD brain. Nevertheless, the amyloid hypothesis is the most widely accepted, given that its deposition is the prime event found in the etiology of AD [9]. The amyloid burden is highly neurotoxic and inflicts other pathological events, including inflammation, tau phosphorylation, oxidative stress, and mitochondrial damage [4]. The ongoing tests on new drug candidates against Aß deposition and tau aggregation show favorable results in animal models but cannot deliver promising effects in clinical trials [9].

Choline acetyltransferase (ChAT) is responsible for the synthesis of the neurotransmitter acetylcholine (ACh) from acetyl-CoA, choline, and ATP, whereas acetylcholinesterase (AChE) tightly controls the hydrolysis of ACh [10]. During AD progression, ChAT activity is significantly decreased in the brain [11]. The reduction in the level of ChAT activity leads to a significant decline in ACh that triggers cholinergic toxicity caused by the overactivity of AChE. The inhibition of AChE helps to restore ACh function and synaptic transmission. Tacrine was the first approved AChE inhibitor by the FDA but was discontinued due to hepatotoxicity [12]. Donepezil, galantamine, and rivastigmine are the current anti-AChE drugs commercialized to reduce cholinergic toxicity. These drugs are capable of ameliorating memory deficit but can only satisfy the limited symptomatic effects of AD.

Diabetes Mellitus is pathophysiologically linked to AD, whereby insulin resistance and hyperinsulinemia related to the central nervous system can lead to cognitive deterioration [13]. According to the meta-analysis of merged scientific results, individuals with type 2 diabetes have a risk of exhibiting AD symptoms at 56% [4]. The reduction in both the levels of insulin and sensitivity of the insulin receptors in the brain can trigger Aß toxicity and tau burdens. Type 3 diabetes describes the correlation between diabetes and AD patients manifesting diabetes-related symptoms [4].

Lipid and protein oxidations are also evident within AD patients’ neurons or brain cell membranes due to the overproduction of reactive immediate species (RIS) [14]. The RIS comprise reactive oxygen species (ROS) and reactive nitrogen species (RNS) that appear from several pathological events, especially mitochondrial dysfunction [4]. During the respiratory chain reaction, an array of redox enzymes in mitochondria transmits electrons to molecular oxygen to generate ROS. The interaction of fusion dynamin-related protein 1 (Drp1) (responsible for mitochondrial dynamics) with Aß and tau will impair mitochondria. The excessive creation of RIS leads to oxidative stress that damages biomolecules and initiates neuronal death [4,14].

The multiple complex toxicities, including endoplasmic reticulum stress and telomerase malfunction, can trigger neuroinflammation and inflammatory proteins that deteriorate cognitive functions [4,15]. Microglial cells play a crucial role in initiating inflammation as a response to the immune system. Oxidative stress, the accumulation of Aß, and other neurotoxicities activate microglia to release cytokines and other inflammatory mediators such as tumor necrosis factor-alpha (TNF-α), interleukin-12 (IL-12), and lipopolysaccharide (LPS) that elicit acute neuroinflammation [4]. Anti-inflammatory and antioxidant drugs are good candidates to reduce immune outrage and oxidative stress.

A few plant genera have been assessed for their neuroprotective capacity. For example, the evidence of the genus *Pistachia* in neuroprotection is based on motor function, behavior, memory impairment, antioxidant, inflammatory markers, neural toxicity, Aß aggregation, and AChE studies in animal models [16]. Their related molecular mechanisms were gathered based on in vitro and in vivo models. Berry fruits such as blueberry, strawberry, mulberry, blackberry, blackcurrant, and bilberry were also reviewed for their neuroprotective effects [17]. Owing to their phytochemicals such as anthocyanin, kaempferol, caffeic acid, catechin, quercetin, and tannin, these bioactive principles are essential in modulating signaling pathways to enhance neurotransmission, neuroplasticity, and cell survival via their anti-inflammatory and antioxidant efficacies.

In this review, a literature survey related to neuroprotection in *Syzygium* was conducted via a bibliographic databases assessment, including PubMed, ScienceDirect, Wiley Online Library, SpringerLink, and Google Scholar. The search was performed within five recent years until March 2022 for all biological activities except for anti-cholinesterase, anti-diabetic, and in vivo memory-related studies. Keywords “cholinesterase”, “anti-cholinesterase”, “AChE”, “diabetes”, “anti-diabetic” “inflammation”, “anti-inflammatory”, “neuroinflammation”, “antioxidant”, “neuroprotection”, “neuroprotective”, “memory”, “cognitive”, “Alzheimer’s”, “phytoconstituent”, and “compound” along with the word “*Syzygium*” were used.

## 3. Ethnobotany and Ethnomedicinal Properties of *Syzgium*

*Syzygium* constitutes a wide range of species that occurs naturally in subtropical and tropical regions of Africa and Madagascar, Asia, and throughout Oceania and the Pacific regions [7,8]. Belonging to the Myrtaceae family, *Syzygium* represents the biggest genus of flowering plants worldwide, encompassing about 1200–1800 species [8]. The largest diversity of *Syzygium* is found in Australia and Southeast Asia. Despite its variety, many species from this region have not been taxonomically classified properly [8]. New species will likely be cataloged from time to time. From medium to large evergreen shrubs and trees, the *Syzygium* genus has a long history of ethnomedicinal usage in Iranian folk medicine and the Ayurveda medicinal system [18,19,20,21]. It is also considered one of the functional foods, given that the leaves and fruits of certain species are consumed for nutrients and health benefits.

Most of the species are edible depending on different plant parts. *Syzygium cumini* has purplish-black fruits containing high nutritional values, which are often commercialized as food products, including jelly, jam, ice cream, and yogurt [22]. *Syzygium aromaticum* is well known for its unopened flower buds that comprise a high amount of clove oil [8]. Their strong aroma enables them to be widely used as spices and flavoring agents for curry and other cuisines in Southern Asia [23]. In Thailand, the young reddish leaves of *Syzygium antisepticum*, having a somewhat astringent and sour taste, are consumed as local vegetables [24]. In Indonesia, the leaves of *Syzygium polyanthum* known as salam, are used in salads (ulam) and cooking the same way as lime leaves are used [25]. In Malaysia, the bright pinkish-red fruits from *Syzygium aqueum* are cut into pieces and eaten with other fruits such as guavas, pineapples, and young mangos as fruit salads (rojak) along with spicy dips. Many young leaves, shoots, and fruits of other *Syzygium* species are consumed, such as *Syzygium jambos*, *Syzygium samarangense*, *Syzygium alliiligneum*, and *Syzygium malaccense* [26].

*S. aromaticum* is ethnopharmacologically important in treating fever, stomachache, colds, flu, diabetes, hypertension, bacterial and fungal infections in the throat, urinary, vaginal tract, skin infections, candidiasis, dyspepsia, and pain and inflammation related to rheumatism [27,28]. Its clove oil is applied as a stimulant and cognitive enhancer in Ayurveda and Iranian traditional practices [18]. Various applications of *S. cumini* have been recorded in the Ayurveda and Unani systems, including the treatment of digestive, diuretic, bronchitis, carminative, wound, antiulcer, antiasthma, antiallergic, and antiscorbutic [19,20,21]. Additionally, the seeds are traditionally exercised to treat memory impairment in India [29]. In Brazil, the leaf infusion from *S. jambos* is traditionally used to treat diabetes [30]. The leaves are decocted for diuretic, rheumatism, and sore eyes reliever, while the seeds are administered to treat diarrhea, dysentery, diabetes, and catarrh. A decoction of the bark of *S. jambos* is applied to relieve asthma and bronchitis [31].

Since the Unani and Ayurveda medical systems, *Syzygium* species holds significant value in traditional medicine to treat ailments related to AD. As mentioned before, the seeds of *S. cumini* are attributed to memory enhancement [29], while its fruit possesses anti-diabetic activity that can benefit Alzheimer’s treatment [32]. Apart from that, *S. jambos* leaves are traditionally used to treat diabetes [33]. The wide range of medicinal properties exerted by *S. aromaticum* also includes anti-diabetic, anti-inflammatory, and cognitive enhancement properties that have been recorded in old manuscripts written by Avicenna [27,28]. The traditional medicinal properties of *Syzygium* species in neuroprotection are indeed well established [8], which has driven many researchers to conduct pharmacological studies in the last decades.

## 4. Neuroprotective Agents from *Syzygium*

### 4.1. Anti-Cholinesterase Activity

Anti-cholinesterase activity remains the primary test used to screen anti-AD drug candidates. Table 1 summarizes the anti-cholinesterase activities reported from *Syzygium* species. They are often tested in vitro based on Ellman’s method using AChE extracted from electric eel and butyrylcholinesterase (BChE) from equine serum. Nonetheless, efforts were made to determine in vitro activity using the parasite *Cotylophoron cotylophorum* [34] and the fruit fly *Drosophila melanogaster* [35]. Ex vivo AChE activity was also measured, revealing no significant activity from *S. cumini* [36]. Apart from that, in vivo AChE and BchE activities were determined in alloxan-induced diabetic rats [37] and scopolamine-induced memory-impaired rats [29]. Both studies showed a significant reduction in enzyme activity.

To our best knowledge, about 10 *Syzygium* species were investigated for their anti-cholinesterase potential. *S. aromaticum* (clove) represents the most studied *Syzygium* species, followed by *S. cumini* for this activity. Not only alcohol extracts were explored, but essential oils were also examined. Methanol and ethanol were widely used to extract *Syzygium* species, while leaves were the most studied plant part for this bioactivity. Eugenol compound from clove demonstrated more potent inhibitory activity than its essential oil and extract, indicating this compound might provide major inhibition from this plant [18]. Darusman et al. reported no significant activity from the leaf extract of *S. cumini* [38], but other studies showed a disagreement. A reduction in cholinesterase activity was demonstrated from the essential oil, polyphenol-rich leaf extract as well as the seed extract of *S. cumini* [22,29,37]. The inhibitory activity observed from essential oils of *Syzygium coriaceum*, *S. cumini*, *S. aromaticum*, and *S. samarangense* proposes their economic importance in therapy and nutrition [22,27,39].

The aqueous extract of *S. jambos* leaves revealed no significant activity [30]. Similarly, the aqueous extract of clove buds exhibited very weak activity with >250 µg/mL of IC_50_ [40], suggesting the unsuitability of water as a solvent for this analysis. Amir Rawa et al. reported the highest number of *Syzygium* species (*Syzygium grande*, *Syzygium lineatum*, *S. jambos*, and *S. polyanthum*) exhibiting above 80% cholinesterase inhibition at 200 µg/mL concentration in one experiment [41]. Among them, *S. polyanthum* leaf extract showed the lowest IC_50_ at 8.28 and 6.54 µg/mL against AchE and BchE, respectively. Further fractionation revealed that polar bioactive constituents (tannins and polyphenols) were accountable for the enzyme inhibition. It is noteworthy that the ethanol bud extract from *S. aromaticum* corrected the AchE rate in cerium chloride (CeCl_3_)-induced memory-impaired mice [42]. This study revealed a clear association between memory and AchE activity, where improved cholinergic neural transmission alleviated the state of memory in mice. Additional studies are required to confirm the bioactivity from other less known species of *Syzygium*, but the potency of inhibiting cholinesterase from this genus is well established.

**Table 1:** Summary of anti-cholinesterase activities exerted from *Syzygium* species.

	Species	Plant Part/Compound	Test	Activity	Reference
1	*Syzygium cumini* (L.) Skeels.	Ethanol leaf extract	In vitro AChE	44.54 µg/mL of IC_50_	[36]
			Ex vivo AChE	No significant effect	
		Leaf essential oil	In vitro AChE	32.9 µg/mL of IC_50_	[22]
		Polyphenol-rich leaf extract	In vitro AChE and BChE	Significant reduction in cholinesterase activities; bound polyphenolic extract showed better inhibitory activity than free polyphenolic extract	[37]
		Polyphenol-rich leaf extract	In vivo AChE and BChE from alloxan-induced diabetic rats	Enzyme activities were significantly reduced after 14 days (400 mg/kg oral dose)	[37]
		Methanol seed extract	In vivo AChE from scopolamine-induced rats	Significant reduction in AChE activity (400 mg/kg oral dose)	[29]
		Leaf extract	In vitro AChE	No significant activity	[38]
2	*Syzygium aqueum* Alston	Methanol leaf extract	In vitro ACHE and BChE	16.04 µg/mL and 13.95 µg/mL of IC_50_, respectively	[43]
3	*Syzygium polyanthum* (Wight) Walp.	Methanol and ethyl acetate extracts from leaves	In vitro ACHE	47.30 and 45.10 µg/mL of IC_50_, respectively	[38]
		Methanol leaf and stem extracts	In vitro ACHE and BChE	>80% inhibition at 200 µg/mL concentration (8.28 and 6.54 µg/mL of IC_50_ in the leaf extract, respectively)	[41]
4	*Syzygium aromaticum* (L.) Merrill and Perry	Methanol, ethyl acetate, and hexane extracts from leaves; methanol bud extract	In vitro ACHE	42.10, 55.9, and 62.05 µg/mL of IC_50_, respectively (leaves); 45.25 µg/mL of IC_50_ (bud)	[38]
		Methanol extract, clove oil, and eugenol	In vitro ACHE and BChE using TLC bioautography	Eugenol (42.44 and 63.51 µg/mL of IC_50_) showed better inhibition than extract (61.5 and 103.53 µg/mL of IC_50_) and oil (49.73 and 88.14 µg/mL of IC_50_), respectively	[18]
		Clove bud essential oil	In vitro ACHE and BChE	1.5 μL/L and 18.2 μL/L of IC_50_, respectively	[27]
		Ethanol extract	HPTLC-densitometry	Showed efficiency in AChE inhibition	[44]
		Ethanol bud extract	In vitro AChE isolated from human erythrocytes	No inhibitory effect	[45]
		Ethanol bud extract	In vitro AChE of parasite *C. cotylophorum*	86.86% inhibition at 0.5 mg/mL after 8 hr exposure	[34]
		Clove oil (eugenol) encapsulated with a nanostructured lipid carrier	In vitro ACHE and BChE from *D. melanogaster* tissue	4.3 and 3.5 mM of IC_50_, respectively	[35]
		Aqueous and hydroalcoholic extract of clove buds	In vitro AChE	253.29 µg/mL of IC_50_ in aqueous extract	[40]
		Clove oil	In vitro AChE from AlCl_3_-induced rats	Significant reduction in AChE activity	[46]
		Ethanol bud extract	In vivo AChE from CeCI_3_-induced memory-impaired rats	Corrected the AChE rate caused by CeCI_3_ toxicity and improved cholinergic neural transmission	[42]
		Eugenol derivatives	In vitro ACHE and BChE	4-Allyl-2-methoxyphenyl-4-ethyl benzoate inhibited AChE with 5.64 µg/mL of IC_50_	[47]
		Isoeugenol	In vitro ACHE	77 nM of IC_50_	[48]
5	*Syzygium antisepticum* (Blume) Merr. and L.M.Perry	Methanol leaf extract; ursolic acid; gallic acid	In vitro ACHE	61.9% at 300 µg/mL concentration; 81.64% at 200 µg/mL concentration; 73.39% at 200 µg/mL concentration	[24]
6	*Syzygium samarangense* (Blume) Merr. and L.M.Perry	Essential oil	In vitro ACHE and BChE	4.83 and 5.69 mg GALAE/g, respectively	[39]
		Dihydrochalcone	In vitro ACHE and BChE	98.5% inhibition at 0.25 mM and 68% inhibition at 0.20 mM, respectively	[49]
7	*Syzygium coriaceum* Bosser and J. Guého	Essential oil	In vitro ACHE and BChE	4.79 and 7.10 mg GALAE/g, respectively	[39]
8	*Syzygium jambos* (L.) Alston	Aqueous leaf extract	In vitro ACHE from homogenized tissue of rat brain	No significant activity	[30]
		Methanol stem and leaf extracts	In vitro ACHE and BChE	>80% inhibition at 200 µg/mL concentration (16.05 and 15.25 µg/mL of IC_50_ from stem extract, respectively)	[41]
9	*Syzygium grande* (Wight) Walp.	Methanol leaf extract	In vitro ACHE and BChE	>80% inhibition at 200 µg/mL concentration	[41]
10	*Syzygium lineatum* (DC.) Merr. and L.M.Perry	Methanol leaf extract	In vitro ACHE and BChE	>80% inhibition at 200 µg/mL concentration (20.69 µg/mL of IC_50_ for BChE)	[41]

### 4.2. Anti-Diabetic Activity

The most common form of diabetes, type 2 diabetes, is generally associated with hyperinsulinemia and insulin resistance. Insulin resistance causes neurodegeneration and impairment in the brain glucose metabolism and cognition, which are also observed in AD patients [4]. Hyperinsulinemia increases tau phosphorylation and Aß accumulation. Furthermore, neuroinflammation, oxidative stress, mitochondrial dysfunction, and advanced glycation products are evident in diabetic and AD patients [4]. Both disorders share similar features; medicinal plants that can stimulate insulin secretion would benefit diabetic as well as AD patients.

Zulcafli et al. extensively reviewed the anti-diabetic potential of eight *Syzygium* species [50]. The review reported that the inhibition of enzymes involving carbohydrate metabolisms such as α-glucosidase, maltase, and α-amylase is the most studied mechanism of action in the anti-diabetic potential of *Syzygium* [50]. Pertaining to type 2 diabetes, the ethanolic seed extract of *S. cumini* was shown to stimulate insulin secretion produced by pancreatic-ß cells in alloxan-induced mild and severely diabetic rabbits [51]. The hydroethanolic extract from *S. cumini* leaves also improved hyperinsulinemia and insulin resistance by modulating ß-cell insulin release in monosodium L-glutamate (MSG)-induced obese rats [52]. Additionally, Sahana et al. demonstrated that a reduction in insulin resistance was evident in 30 newly diagnosed type 2 diabetic patients when *S. cumini* seed powder was administered [53]. The treatment of high-fat diet/streptozotocin (HFD/STZ)-induced diabetic rats with the aqueous seed extract of *S. cumini* at 400 mg/kg decreased the levels of serum glucose, insulin, and other diabetic markers [54].

A study by Shen et al. demonstrated a clear connection between insulin resistance and inflammation in TNF-α-treated FL83B cells [55]. The suppression of c-Jun N-terminal kinase (JNK) inhibited an inflammatory response as the cells were treated with the fruit extract of *S. samarangense*. As a result, insulin resistance induced by TNF-α was alleviated via the activation of phosphatidylinositol-3 kinase–protein kinase B (PI3K–Akt/PKB) signaling [55]. *S. aqueum* leaf extract, on the other hand, reduced glucose levels, increased insulin secretion, and decreased the collagen deposition associated with its anti-inflammatory and antioxidant responses in STZ-induced diabetic rats [56]. It decreased the levels of toll-like receptor 4 (TLR-4), myeloid differentiation primary response 88 (MYD88), TNF receptor-associated factor 6 (TRAF-6), and TNF-α correlated to pancreatic inflammatory cell infiltration. Malondialdehyde, a sensitive biomarker of ROS-induced lipid peroxidation, was also reduced [56].

In another study, vescalagin isolated from *S. samarangense* ameliorated insulin resistance in high-fructose diet-induced hyperglycemic rats [57]. Myricitrin isolated from the *S. malaccense* leaf extract exhibited insulin-like effects by enhancing lipid accumulation, glucose uptake, and adiponectin secretion via the activation of the insulin signaling pathway [58]. The aqueous extract of *Syzygium paniculatum* fruits alleviated hepatic insulin resistance at a 100 mg/kg dose by reducing the blockage of the insulin signaling pathway via the improvement of insulin receptor (IR and IRS-1) function in HFD-induced diabetic rats [59]. In addition, the IR mRNA levels were restored to the control level in type-2 diabetic rats treated with *Syzygium jambolanum* homeopathic remedies, suggesting improvement in insulin secretion [60].

### 4.3. Anti-Inflammatory Activity

Nearly all pathological events, including endoplasmic reticulum stress and autophagy dysfunction, can trigger inflammatory responses in AD [4]. Moroever, insulin resistance and diabetes have been shown to correlate well with inflammation. For example, the bark extract of *S. jambos* improved the insulin receptor substrate-2/protein kinase B/glucose transporter-4 (IRS-2/AKT/GLUT4) insulin signaling pathway in the liver while improving glycemic parameters by suppressing inflammation, oxidative stress, and apoptosis in STZ-induced rats [61]. Inflammation is defined as a physiological defense mechanism by the immune system to combat health hazards, causing pain to occur [4]. It was demonstrated that microglia accumulate in higher quantities near Aß plaques than in the healthy brain. Amyloid plaques and other factors can activate microglia to initiate neuroinflammation [4]. Anti-inflammatory drugs enable the central nervous system (CNS) to impede pain signaling in the brain, therefore, reducing inflammation in AD pathogenesis. Recent anti-inflammatory activities reported from *Syzygium* were summarized in Table 2.

The levels of pro-inflammatory mediators such as IL-6, IL-1β, and TNF-α were generally measured to determine the anti-inflammatory activity (Table 2). The inflammation was induced by a toxic chemical or drug such as alloxan and STZ to stimulate inflammatory diabetes in model rats. LPS- or HFD-induced inflammation in diabetic rats was also conducted to observe the anti-inflammatory potential of *Syzygium*. So far, one study has demonstrated a close correlation between inflammation and memory loss in AD model rats. The memory-related learning ability of Aβ_1-40_-infused AD model rats was improved as pro-inflammatory TNF-α and lipid peroxide (LPO) were suppressed when *S. cumini* seed extract was administered [62]. The leaf, fruit, pulp, and seed extracts of *S. cumini* exerting anti-inflammatory activity suggested that almost all parts are bioactive (Table 2).

*S. malaccense* leaf extract exerted neuroinflammatory protection against LPS-induced neuroinflammation on murine BV-2 microglial cell lines by reducing nitric oxide production [63]. Nitric oxide (NO) is one of the pro-inflammatory mediators released by microglia; reducing the NO levels can minimize immune outrage caused by microglia [4]. Other less studied *Syzygium* species have also been observed to exert anti-inflammatory activities, including *Syzygium caryophyllatum*, *Syzygium mundagam*, *Syzygium calophyllifolium*, and *S. samarangense* (Table 2).

**Table 2:** Summary of anti-inflammatory activities reported from *Syzygium* species.

	Species	Plant Part/Compound	Test	Activity	Reference
1	*S. malaccense* (L.) Merr. and L.M. Perry	Methanol leaf extract	In vitro LPS-induced neuroinflammatory assay on murine BV-2 microglial cells; in vivo croton oil-induced ear edema test	Neuroprotective activity by a reduction in nitric oxide production in vitro; decreased mice ear edema in vivo	[63]
2	*S. cumini*	Methanol fruit extract	In vitro membrane stabilization, egg albumin denaturation, and bovine serum albumin denaturation assays; in vivo murine models of carrageenan, formaldehyde, and PGE_2_ induced paw edema.	Showed inflammatory activities both in vitro and in vivo	[64]
		Betulinic acid	In vivo Fx1A antiserum-induced passive Heymann nephritis (PHN) in Sprague-Dawley rats	Ameliorated mRNA and protein expression of NF-κB, iNOS, TNF-α, Nrf2, HO-1, and NQO1 in the kidney, reducing inflammation	[65]
		Polyphenol-rich leaf extract	In vivo Alloxan-induced diabetic rats	NF-κB and inflammatory cytokines such as TNF-α and IL-1α were regulated	[37]
		Anthocyanins di-glucosides from pulp	In vitro determination of cytokine production in LPS-induced RAW264.7 macrophages	Inhibited pro-inflammatory mediators such as IL-6, IL-1β, and TNF-α	[66]
		Aqueous seed extract	In vivo high cholesterol diet-streptozotocin-induced diabetes in rats	Exhibited significant anti-inflammatory and β-cell salvaging activity via overexpression of PPARγ and PPARα activity and a significant decrease in TNF-α levels when treated with 100, 200, 400 mg/kg/day doses	[67]
		Methanol seed extract	In vitro high glucose (HG) diabetic cardiomyopathy in H9C2 cardiomyoblast cells	HG-induced activation of NF-*κ*B, TNF-*α*, and IL-6 was remarkably reduced	[68]
		Seed extract	In vivo Aβ_1-40_-infused AD model rats	Reduced the levels of Aß burdens and oligomers by suppressing the levels of TNFα and LPO in the corticohippocampal tissues	[62]
3	*Syzygium caryophyllatum* (L.) Alston	Aqueous root extract	In vitro anti-inflammatory test using heat-induced albumin denaturation assay	6.229 µg/mL of IC_50_	[69]
4	*S. aqueum*	Polyphenol-rich leaf extract	In vitro lipoxygenase inhibitor screening assay, membrane stabilizing activity (hypotonic solution-induced hemolysis), and in vivo carrageenan-induced hind-paw edema in rats	Inhibited LOX, COX-1, and COX-2 with higher COX-2 selectivity reduced the extent of lysis of erythrocytes and markedly reduced leukocyte numbers in rats challenged with carrageenan.	[70]
		Leaf extract	In vivo STZ-induced oxidative stress and inflammation in pancreatic beta cells in rats	Significantly decreased levels of TLR-4, MYD88, pro-inflammatory cytokines TNF-α, and TRAF-6 in pancreatic tissue homogenates, which correlated well with minimal pancreatic inflammatory cell infiltration	[56]
5	*Syzygium mundagam* (Bourd.) Chithra	Methanol bark extract	In vivo carrageenin- and egg albumin-induced paw edema, cotton pellet implanted granuloma in rats	Effective anti-inflammation at 200 mg/kg dose	[71]
6	*Syzygium calophyllifolium (Wight) Walp.*	Methanol bark extract	In vivo carrageenin- and egg albumin-induced paw edema, cotton pellet implanted granuloma	200 mg/kg dose significantly reduced the paw edema in carrageenan (96.71%) and egg albumin models (54.24%) compared to the control. Chronic inflammation was also inhibited by up to 70.46%	[72]
7	*S. aromaticum*	Ethanol/water extract	In vivo carrageenan-induced paw edema inflammatory in rats	Pretreatment at different doses (100, 200, and 400 mg/kg) produced a significant (*p* < 0.001) reduction in paw inflammation up to 5 h of carrageenan injection	[73]
		Essential oil	In vivo formalin-induced and carrageenan-induced paw edema inflammation in rats	26.9 ± 2.5 μg/paw of EC_50_	[74]
		Aqueous clove extract	In vivo LPS-induced lung inflammation in mice.	Inhibited matrix metalloproteinases: MMP-2 (15%) and MMP-9 (18%) activity in lung homogenates, reducing inflammation	[75]
		Ethanol extract	In vitro TNF-α induced inflammation in dental pulp stem cells	Prevented the increase in IL-6 levels	[76]
		Eugenol	Cytochrome c reduction assay to measure superoxide anion generation in human neutrophils	Inhibited the generation of superoxide anion by neutrophils via the inhibition of Raf/MEK/ERK1/2/p47phox-phosphorylation pathway	[77]
		Eugenol	In vivo ethanol-induced ulcer in rats	Decreased TNF-α and IL-6 cytokine concentrations responsible for inflammation	[78]
		Essential oil	Isbolographic study using the formalin test in rats	*S. aromaticum* in combination with ketorolac, showed an antinociceptive effect in the treatment of inflammatory pain	[79]
8	*S. samarangense*	Polyphenol vescalagin	In vivo methylglyoxal-induced inflammation in diabetic rats	The pancreatic levels of NF-κB, ICAM-1, and TNF-α protein, were reduced	[80]
		Lyophilized fruit powder	In vivo STZ-induced pancreatic beta cells apoptosis in rats	Pancreatic ß-cell apoptosis was alleviated with significantly down-regulated cleaved caspase-3 and Bax and upregulated Bcl-2 and Bcl-xl protein expression	[81]
9	*S. polyanthum*	Leaf extract	In vivo coronary artery ligation-induced myocardial infarction in rats	Reduced levels of C-reactive protein (CRP) and myeloperoxidase (MPO) in the rats started from day 4 after the induction of myocardial infarction.	[82]
10	*S. jambos*	Bark extract	In vivo streptozotocin-induced inflammation in diabetic rats	Significantly reduced TNF-α and increased IL-10 (*p* < 0.05) in pancreatic tissues	[61]

### 4.4. Antioxidant Activity

Oxidative stress is highly apparent when redox circuitry is disrupted, and macromolecular damage occurs, leading to an imbalance in the pro-oxidant and antioxidant levels [83]. The overproduction of RIS, such as hydrogen peroxide (H_2_O_2_), hydroxyl radical (HO), and NO observed in the AD brain, can trigger severe oxidative stress. Various factors can contribute to excessive RIS, including mitochondrial dysfunction, high levels of cytochrome oxidase, and Aß peptide chelation by redox-active metal ions [4,83]. Moreover, the downregulation of the expression and activity of antioxidant enzymes such as dehydrogenase complexes is evident in AD, causing biomolecular damage (lipids, proteins, and DNA) and neuronal death [4]. Antioxidants help scavenge free radicals and balance the production of RIS to reduce oxidative damage [84].

*Syzygium* is undeniably a great source of antioxidant agents due to the presence of polyphenols, tannins, and flavonoids [84]. The evidence of antioxidant activities from *Syzygium* species was summed up in Table 3. Compared to other biological activities, the antioxidant potential is the most comprehensively explored in various *Syzygium* species, including less studied *Syzygium cymosum*, *S. paniculatum*, and *S. caryophyllatum*. They not only scavenge free radicals but also provide protective effects against other toxicities. For example, CeCl_3_-induced neurotoxicity in the brains of rats was improved by the antioxidant capacity of *S. aromaticum* to restore RIS levels, which alleviated the cholinergic neural transmission and the state of memory in mice [42]. In another study, the ability of antioxidants to maintain genomic stability and slow down aging was demonstrated in the *Caenorhabditis elegans* nematode model [85]. The essential oil of *S. aromaticum* exerted antioxidant potential by inducing the expression of SOD-3 or GST-4 (antioxidant enzymes) and DAF-16/FOXO nuclear translocation from the cytoplasm to promote longevity in *C. elegans* [85].

*S. aromaticum* (clove) is indeed the most studied *Syzygium* species for its antioxidant capacity. Its economically important essential oil source has brought many interests toward its applications. Alfikri et al. reported that clove produced the best essential oil ingredient at the flowering stage and the most efficient source of antioxidants when the trees are young. [86]. Meanwhile, Teles et al. demonstrated that eugenol (the primary compound of clove oil) exhibited higher antioxidant activity than its essential oil [87]. Various plant parts of the clove have been examined, especially its bud essential oil (Table 3). As for *S. cumini* and *S. malaccense*, their dried peel powders showed higher phenolic compounds, anthocyanin content, and antioxidant activity than their freeze-dried extracts, which can be pharmacologically relevant to their food applications [88].

**Table 3:** Summary of plant parts or compounds examined for antioxidant activity from *Syzygium* species.

	Species	Plant Part/Compound	Reference
1	*S. cumini*	Leaf	[36,37,89]
		Fruit	[64,88,90]
		Bark	[91]
		Polyphenol-rich extract	[92,93]
		Seed kernels powder	[94]
2	*S. polyanthum*	Leaf	[38]
3	*S. aromaticum*	Flower	[86]
		Bud	[42,95]
		Bud essential oil	[27,86,87,96,97,98]
		Eugenol	[87]
		All parts	[99]
4	*S.* *antisepticum*	Leaf	[24]
		Gallic acid, myricitrin, and quercitrin	[24]
5	*S. caryophyllatum*	Leaf	[100]
		Fruit	[100,101]
		Fruit pulp healthy snack	[102]
6	*Syzygium paniculatum* Gaertn.	Leaf	[103]
		Fruit	[104]
		Volatile oil from the aerial part	[105]
7	*S. malaccense*	Leaf	[63,88,106]
		Myricetin derivatives	[107]
8	*S. aqueum*	Stem	[108]
		Bark	[108]
9	*S. polyanthum*	Leaf	[109]
10	*S. jambos*	Fruit	[110]
		Bark	[61]
11	*S. samarangense*	Vescalagin	[80]
12	*Syzygiumcymosum* (Lam.) DC.	Leaf	[111]

### 4.5. In Vivo Neuroprotection Studies

The roles of *Syzygium* in neuroprotection were validated in animal models (Table 4). Aß_1-40_ and Aß_1-42_ are the major amyloid species observed in the buildups of AD brains. A study by Hossain et al. investigated the amyloid deposition and neurobehavioral symptoms in Aß_1-40_-infused AD rats when the seed extract of *S. cumini* was administered [62]. The extract elevated the memory-related learning ability by suppressing the levels of TNF-α and LPO, suggesting that anti-inflammatory and antioxidative actions took place in reducing the amyloid deposits [62]. In another study, the clove oil combined with exercise increased the levels of antioxidant-related enzymes peroxiredoxin-6 (PRDX6) and general control of amino acid synthesis 5-like 1 (GCN5L1) in Aß_1-42_-infused spatial memory-impaired AD rats. This observation specifies the importance of *S. aromaticum* oil as an antioxidant agent [28].

Indeed, oxidative stress and its impacts on AD pathogenesis have been largely concentrated in several other cognitive-impaired rat models. Significant improvement in the neurological deficit score was observed via the clove oil administration by which endogenous antioxidants such as SOD, CAT, and GSH were enhanced [96]. In aluminium chloride (AlCl_3_)-induced neurotoxic rats, the bud extract restored the brain ions homeostasis and oxidative level [46]. The bud extract of *S. aromaticum* also restored oxidative stress biomarkers, reduced AChE activity, and improved memory impairment in CeCl_3_-induced AD rats [42]. An improvement in the symptoms of retracted neurons with condensed chromatin undergoing necrosis or apoptosis and vacuolated space was observed in the brain tissues.

In addition, the seed extract from *S. cumini* ameliorated the learning-related memory of hypoxia-induced old male rats based on the reduction in the level of LPO [112]. The oxidative injury triggered by hypoxia caused the presence of high microglial cells around the hippocampus as a result of immune outrage. The antioxidative defense of the seed extract on the hypoxic group decreased the level of LPO that reduced inflammation caused by microglial invasion in the corticohippocampal brain tissue [112]. As a result, the brain cells retained their normal cellular structures with less swelling and rupture.

HFD-induced obesity can result in insulin resistance that can enhance pathophysiological processes related to AD in the brain [113]. The fruit of *S. malaccense* improved the learning and memory tasks of HFD-induced cognitive-impaired rats by alleviating AKT signaling in the hippocampus, preventing the activation of GSK3-ß, and lowering tau phosphorylation [114]. It stimulated peripheral insulin activity and brain antioxidant enzyme activities, promoting an anti-AD effect in this diabetic model. In alloxan-induced diabetic rats, the polyphenol-rich extract of *S. cumini* leaves showed a neuroprotective effect via suppression of the cholinesterase activity and reduction in the lipid peroxidation and hydroperoxide concentration [37]. This study justified the presence of AD symptoms in a diabetic model.

Kumaran et al. developed chronic hypoperfusion (CCH)-induced rats via a permanent occlusion of bilateral common carotid arteries (POBCCA) surgery to evaluate the effect of *S. aqueum* leaves on dementia [43]. This plant extract improved short- and long-term recognition memories and spatial learning based on the behavioral tests, including automated open field test, novel object recognition (NOR), and a Morris water maze (MWR) test. Additionally, the plant extract exhibited good anti-AChE and anti-BChE activities, although the antioxidant and anti-inflammatory parameters were not determined.

**Table 4:** Reported in vivo studies related to neuroprotection or aging from *Syzygium* species.

	Species	Plant Part/Compound	Test	Activity	Reference
1	*S. cumini*	Seed extract	Eight-arm radial maze task for learning-related memory	Improved learning-related memory through the antioxidative defense by a reduction in corticohippocampal levels of lipid peroxide	[112]
		Seed extract	Aß_1-40_-infused AD model rats	Significantly increased the memory-related learning ability of the AD model rats with reductions in the levels of corticohippocampal Aβ_1-40_-burden and Aβ_1-40_-oligomers, and increased the levels of brain cognition and memory-related proteins, including BDNF, TrKB, PSD-95 and SNAP-25	[62]
2	*S. aqueum*	Methanol leaves	POBCCA surgery in rats	Improved short- and long-term recognition memory in NOR test, improved spatial learning in MWM test at 200 mg/kg dose	[43]
3	*S. aromaticum*	Aqueous bud extract	AlCl_3_-induced neurotoxic rats	Restored the parameters (Al, Ca^2+^, MDA, nitrite/nitrate, Mg^+^, Na^+^, GSH, GPx) to the near-normal levels, significantly normalized expression of the SOD1 gene	[46]
		Clove oil	Amyloid_1-42_-induced spatial memory-impaired rats	Improved spatial memory in Shuttle box test and apoptosis, PRDX6, and GCN5L1 levels were recovered through swimming training and clove consumption	[28]
		Clove oil	MCAO-stroke-induced rats	The pre-treated and post-treated groups with clove oil showed improvement in neurological deficit score	[96]
		Ethanol bud extract	CeCI_3_-induced memory-impaired mice	Symptoms of retracted neurons with condensed chromatin undergoing necrosis or apoptosis and vacuolated space were alleviated, which improved the state of memory in mice	[42]
		Clove essential oil	*C. elegans* model	Extended lifespan and promoted production and health of *C. elegans* by inducing DAF-16/FOXO nuclear translocation from the cytoplasm and causing apoptosis of germ cells in ACEP-1 and DAF-16	[85]
4	*S. malaccense*	Freeze-dried fruit	HFD-induced cognitive impaired rats	Improved AKT signaling in the hippocampus that prevented the activation of GSK3-β, lowered tau phosphorylation, and improved brain antioxidant enzyme activities.	[114]

## 5. Bioactive Phytoconstituents

In general, *Syzygium* species are rich in flavonoids, tannins, phenols, steroids, as well as alkaloids [7,50] (Table 5). The polyphenol-rich extract of *S. cumini* has shown potent anti-cholinesterase, anti-inflammatory, and antioxidant activities [37,92,93]. Betulinic acid from this plant ameliorated mRNA and protein expressions such as NF-κB, iNOS, TNF-α, Nrf2, HO-1, and NQO1 to reduce inflammation in Fx1A antiserum-induced passive PHN in Sprague-Dawley rats [65]. It restored antioxidant activities and malondialdehyde levels effectively. The cholinesterase inhibitor, eugenol from *S. aromaticum*, inhibited the generation of superoxide anion via the suppression of the Raf/MEK/ERK1/2/p47phox-phosphorylation pathway and decreased cytokine concentrations attributed to its antioxidant and anti-inflammatory activities [18,77,78]. Its derivative, isoeugenol, exhibited potent inhibition against AChE, α-amylase, and α-glucosidase at 77.00, 411.5, and 19.25 nM of IC_50_, respectively [48].

Vescalagin isolated from *S. samarangense* ameliorated insulin resistance in high-fructose diet-induced hyperglycemic rats and reduced the pancreatic levels of NF-κB, ICAM-1, and TNF-α proteins [57,80]. Its antioxidant capacity helped to elevate the antioxidant contents in methylglyoxal (MG)-induced diabetic rats. 2′,4′-dihydroxy-6′-methoxy-3′,5′-dimethyl-dihydrochalcone from this species inhibited 98.5% at 0.25 mM and 68.0% at 0.20 mM against AChE and BChE, respectively [49]. The antioxidant agents, myricetin derivatives (with 77% of myricitrin) from *S. malaccense* exhibited insulin-like effects by enhancing lipid accumulation, glucose uptake, and adiponectin secretion via the activation of the insulin signaling pathway [58]. In *S. antisepticum*, ursolic acid and gallic acid showed fair inhibition against AChE [24]. Myricitin and quercitrin isolated from this species suppressed the production of ROS and catalase in HEK-293 cells [24]. An anti-AChE bioassay-guided isolation of *S. jambos* afforded two anacardic acid derivatives, 6-heptadeca-8Z,11Z,14Z-trienyl salicylic acid (SB-202742) and 6-heptadeca-9Z,12Z-dienyl salicylic acid (anacardic acid C) [115]. Both compounds inhibited AChE at 0.54 and 2.4 µM of IC_50_, respectively.

A few molecular docking studies have been performed investigating the effect of compounds from *Syzygium* against cholinesterase and diabetic enzymes. (*E*)-β-caryophyllene, one of the major compounds of *S. cumini*, revealed the best docking scores at −6.75, −5.61, and −7.75 kcal/mol for AChE, α-amylase, and α-glucosidase, respectively, implying its ability to form an enzyme–ligand complex [22]. Sharmeen Jugreet et al. identified ß-pinene and (E)-ß-ocimene from the essential oils of *S. samarangense* and *S. coriaceum*, respectively [39]. These compounds showed molecular docking scores within a range of −3.8 to −6.4 kcal/mol against AChE, BChE, α-amylase, and α-glucosidase. SB-202742 and anacardic acid C from *S. jambos* formed molecular interactions with residues TRP 84, GLY 118, and GLY 119 of *Torpedo californica* AChE [115]. An additional polar H-bond with ALA 201 and a π–alkyl non-polar interaction with TYR 121 from anacardic acid C were observed, justifying its superior activity than SB-202742 due to the conformational flexibility of its saturated alkyl chain.

**Table 5:** Reported bioactive principles for neuroprotection from *Syzygium* species.

	Compound Name	Chemical Structure	Biological Activity	Reference
1	Betulinic acid (in powder)	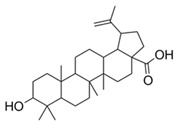	Anti-inflammatory and antioxidant	[65]
2	Eugenol (in MeOH)	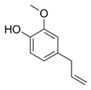	Anti-cholinesterase, anti-inflammatory, and antioxidant	[18,77,78]
3	Isoeugenol (in EtOH)	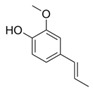	Anti-cholinesterase, and anti-diabetic	[48]
4	Vescalagin (in lyophilized powder)	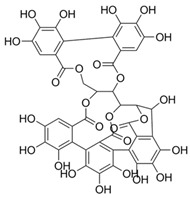	Anti-inflammatory, antioxidant, and anti-diabetic	[57,80]
5	2′,4′-Dihydroxy-6′- methoxy-3′,5′-dimethyl-dihydrochalcone	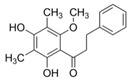	Anti-cholinesterase	[49]
6	Ursolic acid (in DMSO, tween 20, or MeOH)	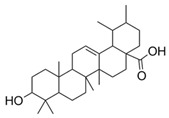	Anti-cholinesterase	[24]
7	Gallic acid (in DMSO, tween 20, or MeOH)	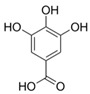	Anti-cholinesterase	[24]
8	Myricitin (in DMSO, tween 20, or MeOH)	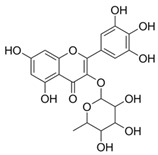	Antioxidant and anti-diabetic	[24,58]
9	Quercitin (in DMSO, tween 20, or MeOH)	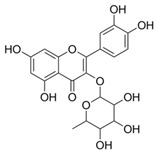	Antioxidant	[24]
10	6-Heptadeca-8*Z*,11*Z*,14*Z*-trienyl salicylic acid (in DMSO)	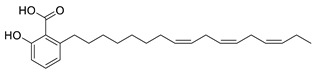	Anti-cholinesterase	[115]
11	6-Heptadeca-9*Z*,12*Z*-dienyl salicylic acid (in DMSO)	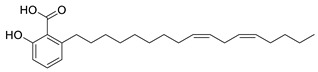	Anti-cholinesterase	[115]
12	(*E*)-β-Caryophyllene (in essential oil)	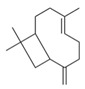	Anti-cholinesterase and anti-diabetic	[22]
13	ß-Pinene (in essential oil)	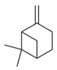	Anti-cholinesterase and anti-diabetic	[39]
14	(*E*)-ß-Ocimene (in essential oil)	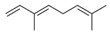	Anti-cholinesterase and anti-diabetic	[39]

DMSO: dimethyl sulfoxide; EtOH: ethanol; MeOH: methanol.

## 6. *Syzygium aromaticum*

The flowering bud, known as clove, from *S. aromaticum* can undoubtedly be found in almost every household. This common spice is largely used as a flavoring or as preservative [116]. Clove, historically, originated from the “Spice Island” of Maluku in Eastern Indonesia but is also cultivated in other parts of Asia, including India, Malaysia, and Sri Lanka [117]. Madagascar, Tanzania, and the West Indies also harvest cloves in high quantities. This aromatic spice plant is also known as *Eugenia caryophyllus*, *Myrtus caryophyllus*, *Jambosa caryophyllus*, *Caryophyllus aromaticus*, and *Caryophyllus silvestris* [118]. Due to different harvest seasons across countries with different climates, clove is available throughout the year. It requires well-distributed rainfall with high humidity and temperatures around 25 to 35 °C.

The clove bud contains almost 18% of essential oil, consisting of eugenol (the main bioactive constituent), eugenol acetate, and β-cariofilen [119]. Clove is indeed rich in phenolic compounds, including flavonoids, hydroxybenzoic acids, hydroxyphenyl propens, hydroxycinnamic acids, and gallic acid derivatives such as hydrolyzable tannins [117]. Flavonoids such as quercetin and kaempferol and phenolic acids such as ferulic, caffeic, ellagic, and salicylic acids are among the compounds reported in clove. These phenolic compounds are accountable for various biological activities such as the anti-microbial and antioxidant properties that have positioned clove as one of the best natural food preservatives among other spices.

Clove receives the most attention for the *Syzygium* species, and its pharmacological studies are constantly reported. Its pre-clinical and clinical studies in AD remain to be conducted. However, its enhancement of memory and cognitive functions has been immensely demonstrated in animal models. The mechanisms of action in the anti-AD effect reported from the clove are via its antioxidant capacity, protection against neuroinflammation, restoration of cholinergic and mitochondrial functions, and hypocholesterolemic activity. It increased the levels of antioxidant-related enzymes such as PRDX6 and GCN5L1 and the potential mediators of oxidative pathways such as SIRT1 to maintain oxidative balance [28,95,120]. The clove oil restored mitochondrial function by increasing the activities of the mitochondrial respiratory enzyme complex (I–IV) [121]. The brain cholinesterase activity and total cholesterol level were also significantly decreased, which enhanced cholinergic transmission and reversed amnesia [122]. This improvement in the learning and memory parameters was confirmed by the decreased levels of TL and TRC and the increased values of SDL.

## 7. Conclusions

This review has highlighted the pharmacological importance of *Syzygium* species in neuroprotection, which justified their ethnomedicinal relevance in relation to memory and cognitive enhancement. Despite much interest and rigorous scientific research toward this genus, *S. aromaticum* and *S. cumini* have gained the most attention due to their widely medical applications in the Ayurveda, Unani, and Iranian medicinal systems. These two species are well documented from both ethnomedicinal and pharmacological perspectives. The lack of traditional practices for the other species in other traditional systems such as Western, Southern, Central African, and Australian Aboriginal medicinal systems concerning memory and cognitive functions may have influenced the current pharmacological studies to concentrate on particular *Syzygium* species only. Other species, including Western and Australian species, remain largely unstudied. There is an urge to conduct more neuroprotective studies toward less studied species, although their ethnobotanical records seem inadequate.

In short, *S. aromaticum* and *S. cumini* deliver excellent neuroprotective effects against AD. There were a fair number of studies for *S. jambos*, *S. malaccense*, *S. samarangense*, and *S. aqueum* in neuroprotection, and these are worthy of further investigation. Eugenol represents the most studied compound in the *Syzygium* genus. As far as we are concerned, recent studies were more inclusive toward in vitro and in vivo models, with only one clinical study involving 30 newly diagnosed type 2 diabetic patients to observe the efficacy of *S. cumini* seed powder. Other species are far from getting tested for human trials, but the in vivo evidence gathered for *S. aromaticum* and *S. cumini* has proved that these species require clinical studies in the future. Nearly all of the studies examined *Syzygium* species in the form of extracts, which lacked the compound isolation part. Partially purified extracts such as essential oils and polyphenol-rich extracts were assessed, but the bioactive components remain unidentified. Furthermore, only two or three in-silico studies were performed to observe the molecular interactions between isolated compounds and protein targets. Future studies involving isolation, in vivo testing of bioactive compounds, and in silico molecular characterization are essential as broader research areas could be explored.

Correlations between diabetes, inflammation, and oxidative stress are well established, where the suppression of inflammation, pro-inflammatory cytokines, and ROS production stimulates the insulin signaling pathway to promote insulin secretion when *Syzygium* extracts are administered. Additional studies are needed that measureme brain-cognition parameters, memory-related proteins, and the levels of AChE activity in diabetic rat models. Similarly, in memory-impaired rat models, assessing the levels of diabetic parameters and AChE activity is warranted. Incorporating the parameters of all four activities into one experiment or animal model may help to understand their pathophysiological relations better. Overall, the molecular mechanisms of *Syzygium* species in neuroprotection involve the inhibition of AChE to correct cholinergic transmission, the suppression of pro-inflammatory mediators, oxidative stress markers, and ROS production and an enhancement of antioxidant enzymes, the restoration of brain ions homeostasis, the inhibition of microglial invasion, the modulation of ß-cell insulin release, and an enhancement of lipid accumulation, glucose uptake, and adiponectin secretion via the activation of the insulin signaling pathway. It is noteworthy that the *Syzygium* species stand out as functional foods due to their edible parts and nutritional benefit. The pharmacological values of their essential oils, fruits, and leaves in commercial exploitation deserve more recognition; additional studies are warranted focusing on the seeds, stems, and flowers to utilize the genus for the prevention of AD fully.

## Data Availability

Not applicable.
